# Notch Signaling Mediates Skeletal Muscle Atrophy in Cancer Cachexia Caused by Osteosarcoma

**DOI:** 10.1155/2016/3758162

**Published:** 2016-06-09

**Authors:** Xiaodong Mu, Rashmi Agarwal, Daniel March, Adam Rothenberg, Clifford Voigt, Jessica Tebbets, Johnny Huard, Kurt Weiss

**Affiliations:** ^1^Department of Orthopaedic Surgery, University of Texas Health Science Center at Houston, Houston, TX 77030, USA; ^2^Center for Regenerative Sports Medicine, Steadman Philippon Research Institute, Vail, CO 81657, USA; ^3^Cancer Stem Cell Laboratory, Department of Orthopaedic Surgery, University of Pittsburgh, Pittsburgh, PA 15213, USA; ^4^Department of Orthopaedic Surgery, Lenox Hill Hospital, New York, NY 10075, USA

## Abstract

Skeletal muscle atrophy in cancer cachexia is mediated by the interaction between muscle stem cells and various tumor factors. Although Notch signaling has been known as a key regulator of both cancer development and muscle stem cell activity, the potential involvement of Notch signaling in cancer cachexia and concomitant muscle atrophy has yet to be elucidated. The murine K7M2 osteosarcoma cell line was used to generate an orthotopic model of sarcoma-associated cachexia, and the role of Notch signaling was evaluated. Skeletal muscle atrophy was observed in the sarcoma-bearing mice, and Notch signaling was highly active in both tumor tissues and the atrophic skeletal muscles. Systemic inhibition of Notch signaling reduced muscle atrophy.* In vitro* coculture of osteosarcoma cells with muscle-derived stem cells (MDSCs) isolated from normal mice resulted in decreased myogenic potential of MDSCs, while the application of Notch inhibitor was able to rescue this repressed myogenic potential. We further observed that Notch-activating factors reside in the exosomes of osteosarcoma cells, which activate Notch signaling in MDSCs and subsequently repress myogenesis. Our results revealed that signaling between tumor and muscle via the Notch pathway may play an important role in mediating the skeletal muscle atrophy seen in cancer cachexia.

## 1. Introduction

Cachexia is a clinical condition characterized by weight loss, muscle atrophy, fatigue, and weakness in an individual who is not trying to lose weight. The metabolic milieu of cachexia is defined by the progressive decreases of skeletal muscle and adipose tissue and negative protein balance. While cachexia may accompany a number of diseases (e.g., renal failure, COPD, AIDS, and tuberculosis), it frequently occurs in patients with cancer, wherein it is referred to as cancer-associated cachexia (CAC). CAC is a prevalent and debilitating comorbidity of malignancy. CAC is present in over 50% of oncology patients at the time of death and is the immediate cause of death in around 30%. Although Hippocrates wrote about cachexia in antiquity, it remains a clinical problem in dire need of a solution: there are no management strategies or pharmacologic adjuvants that effectively treat or prevent cancer cachexia [1–4].

Cachexia is distinguished from conditions of decreased caloric intake such as anorexia or starvation, in which muscle mass is generally spared [5, 6]. Starvation-associated wasting can be ameliorated by caloric replacement or hyperalimentation, but cachexia is refractory to nutritional support. This may be due to the systemic inflammation of cachexia. There is overproduction of inflammatory cytokines such as tumor necrosis factor-*α* (TNF-*α*) and interleukin-1 (IL-1) in response to chronic systemic pathology, which results in the dysregulation of muscle homeostasis and a catabolic state [5–7]. Inflammatory cytokines have been shown to inhibit myogenic differentiation through the activation of NF-*κ*B [8–10], a pathway known to play a role in muscular dystrophies and inflammatory myopathies [9–12]. Interestingly, close crosstalk between NF-*κ*B and Notch signaling in the regulation of tumor development and metastasis has been reported [13–15].

Notch signaling is involved in the preservation of stem cell quiescence and the maintenance of a stem cell pool in skeletal muscle, helping to keep stem cells in an undifferentiated state [16–18]. Thus, Notch signaling functions as a repressor of myogenesis, and sustained activation of Notch in muscle stem cells has an adverse effect on muscle regeneration [19–23]. Constitutive activation of the Notch1 Intracellular Domain (NICD) in muscle cells results in impaired skeletal muscle regeneration, as well as an increased number of undifferentiated Pax7+ stem cells (satellite cells) [24]. A recent study of pancreatic cancer-associated muscle atrophy demonstrated enrichment of Pax7+ stem cells in skeletal muscle, which is associated with impaired myogenic potential and reduced myotube fusion [1]. Based on these observations, we hypothesized that Notch signaling might play a role in mediating the skeletal muscle atrophy present in CAC.

Sarcoma encompasses a diverse group of malignancies that arise from cells of mesenchymal origin. Although sarcoma represents only 1% of new cancer diagnoses, it accounts for 2% of cancer deaths. Fifty percent of patients with soft tissue sarcoma develop fatal pulmonary metastatic disease. The outlook for these patients is abysmal: they are considered to be incurable and have a median survival of approximately twelve months [25–35]. Because sarcomas arise in tissues such as muscle, bone, cartilage, and adipose, sarcoma patients not only face the morbidity imparted by the disease itself, but also often experience significant musculoskeletal impairment secondary to aggressive surgical treatment ranging from tumor removal to limb amputation. This musculoskeletal morbidity leaves sarcoma patients uniquely susceptible to the debilitating effects of CAC; however, virtually nothing is known regarding the mechanisms of sarcoma-associated cachexia (SAC).

In this study, a sarcoma-carrying mouse model was established, utilizing the murine osteosarcoma cell line K7M2. K7M2 has high metastatic potential and has previously been shown to feature increased Notch signaling when compared with nonmetastatic osteosarcoma cells [36]. The level of Notch signaling was studied in both the tumors and the atrophic skeletal muscles of the mice.* In vitro* coculture of K7M2 cells with muscle-derived stem cells (MDSCs) isolated from normal wild-type (WT) mice without cancer was performed to determine if activated Notch signaling can be transferred from tumor cells to muscle cells and if the myogenic potential of muscle cells could be altered. Additionally, because exosomes have been recognized as important to intercellular communication among tumor cells [37], the potential role of exosomes in remotely delivering Notch-activating factors from tumor cells to muscle cells was evaluated. Finally, because TNF-*α* is known as a key mediator of muscle atrophy in cancer cachexia [38–41] and crosstalk between the TNF-*α* and Notch pathways has been described in cancer development and metastasis [14, 15, 42], we also investigated the potential of TNF-*α* to mediate Notch activation in muscle cells.

## 2. Materials and Methods

### 2.1. Animals and Osteosarcoma Cell Lineages

Wild-type (WT) mice (C57BL/6J) were obtained from Jackson Laboratories (Bar Harbor, ME) and used for the isolation of muscle-derived stem cells (MDSCs). SCID/beige mice (CB17.Cg-*Prkdc* ^*scid*^ *Lyst* ^*bg-J*^/Crl, female, 4-week-old) were obtained from Charles River and used for experiments on cancer cachexia. At least six mice were used in each experimental sample group. All procedures were approved by the Institutional Animal Care and Use Committee (IACUC) at the University of Pittsburgh. Murine osteosarcoma cell lineages K7M2 and K12 used in this study were the generous gift of Drs. Lee Helman and Chand Khanna at the National Cancer Institute. K7M2 and K12 are related murine osteosarcoma cell populations with differing metastatic potentials: K7M2 is highly metastatic to the lung but K12 is virtually nonmetastatic [43]. K7M2 cells and K12 cells were cultured with proliferation medium [PM, DMEM with 10% FBS and 1% penicillin-streptomycin (P/S) antibiotics].

### 2.2. Transplantation of Osteosarcoma Cells

K7M2 cells were locally injected into the right hindlimbs of 4-week-old SCID/beige mice; the cortex of the proximal tibia was punctured with a 30 g needle, and cells were injected into the intramedullary canal (2.0 × 10^5^ cells/per mouse). Osteosarcoma tumor development was then permitted, and muscle tissues were collected for study six weeks after cell transplantation.

### 2.3. Stem Cell Isolation from Skeletal Muscle

Muscle-derived stem cells (MDSCs) were isolated from the skeletal muscle of WT mice (4-week-old) using the modified preplate technique [44]. Mice were sacrificed in a carbon dioxide chamber followed by cervical dislocation according to the IACUC protocol. The cells were cultured in the growth medium [GM: DMEM supplemented with 20% Fetal Bovine Serum (FBS), 1% P/S antibiotics, and 0.5% chick embryo extract (CEE)] at 37°C in 5% CO_2_.

### 2.4. Cell Coculture Experiment, Myogenesis Assay, and Notch Inhibition

Cell coculture was conducted with a transwell system (Corning Transwell) illustrated in Figure 5(a), with a cell nonpermeable filter (0.4 *μ*m). MDSCs (20,000/well in 12-well plate) were cultured in the lower chamber, while the same number of K7M2 or K12 cells was cultured in the upper chamber to determine the influence of osteosarcoma cells on the expression of Notch genes and myogenesis of MDSCs. A control group was provided by MDSCs cocultured with MDSCs, themselves. The *γ*-secretase inhibitor DAPT (N-[N-(3,5-difluorophenacetyl-L-alanyl)]-S-phenylglycine t-butyl ester; Calbiochem) (10 *μ*M in DMSO) was added to the MDSCs cocultured with K7M2 cells to observe the effect of Notch inhibition on myogenesis. Cell coculture was performed in growth medium for two days, with and without DAPT treatment. Then the upper chambers were removed and the medium was switched to myogenic differentiation medium (DM, DMEM supplemented with 2% Horse Serum and 1% P/S antibiotics) for an additional 2 days. Progression of myogenesis of MDSCs was then tracked by immunostaining of the fixed cells with antibody to fast-myosin heavy chain (f-MHC) (Sigma).

### 2.5. Exosome Isolation and Treatment of Muscle-Derived Stem Cells (MDSCs)

K7M2 cells were plated at 60% confluence in plastic flasks and cultured for 2 days. Exosome isolation was performed with the “Total Exosome Isolation Reagent (from cell culture media)” kit (Life Technologies), as instructed. Briefly, 10 mL of cell culture media was harvested and centrifuged at 2000 ×g for 30 minutes to remove cells and debris. The reagent was added to the cell-free culture media (1 : 2), and the solution was incubated overnight at 4°C. The precipitated exosomes were recovered by standard centrifugation at 10,000 ×g for 60 min. The pellet was then added to 10 mL of fresh culture medium for the treatment of MDSCs.

### 2.6. *In Vivo* Notch Inhibition

MK-0752 (Merck) is a potent *γ*-secretase inhibitor that has been used in clinical trials to inhibit Notch activity in tumor development [45, 46]. In order to observe the effect of Notch inhibition on cancer cachexia in the mice, low doses of MK-0752 (50 mg/kg) [47] (5 mg/mL in 10% DMSO) were injected via an intraperitoneal (IP) route 3 times per week, starting two weeks after K7M2 cell injection. MK-0752 injections were continued for 4 weeks. Mice receiving the vehicle (10% DMSO) served as a control.

### 2.7. mRNA Analysis with Semiquantitative Reverse Transcriptase-PCR

Total RNA was obtained from cells or frozen tissues using the RNeasy Mini Kit (Qiagen, Inc., Valencia, CA) according to the manufacturer's instructions. Reverse transcription was performed using the iScript cDNA Synthesis Kit (Bio-Rad Laboratories, Inc., Hercules, CA). The primer sequences are as follows: GAPDH (Forward: TCCATGACAACTTTGGCATTG; Reverse: TCACGCCACAGCTTTCCA); Notch1 (Forward: GCCGCAAGAGGCTTGAGAT; Reverse: GGAGTCCTGGCATCGTTGG); Hes1 (Forward: CCAGCCAGTGTCAACACGA; Reverse: AATGCCGGGAGCTATCTTTCT); TNF-*α* (Forward: GATTATGGCTCAGGGTCCAA; Reverse: CTCCCTTTGCAGAACTCAGG); and Klotho (Forward: CCCAAACCATCTATGAAAC; Reverse: CTACCGTATTCTATGCCTTC). PCR reactions were performed using an iCycler Thermal Cycler (Bio-Rad Laboratories, Inc.). The cycling parameters used for all primers were as follows: incubation of the reaction mix at 95°C for 10 minutes, PCR, 40 cycles of 30 seconds at 95°C for denaturation, 1 minute at 54°C for annealing, and 30 seconds at 72°C for extension. Products were separated and visualized on a 1.5% agarose gel stained with ethidium bromide. All data were normalized to the expression of GAPDH (glyceraldehyde 3-phosphate dehydrogenase).

### 2.8. Histology

Tissue sections of skeletal muscles or tumors were fixed with 4% formalin (10 min) and rinsed with PBS. For Masson Trichrome staining, sections were incubated in Weigert's iron hematoxylin working solution for 10 min and then rinsed under running water for 10 min. Slides were transferred to Biebrich scarlet-acid fuchsin solution for 15 min, followed by incubation in aniline blue solution for another 5 min. Slides were then rinsed, dehydrated, and mounted. For hematoxylin and eosin (H&E) staining, sections were incubated for 5 min in hematoxylin solution prior to counterstaining with eosin. For immunofluorescent staining, the frozen tissue sections were fixed with 4% formalin and the primary antibodies to Pax7 (DHSB) and Notch3 (Santa Cruz) were applied at 1 : 100~1 : 200. All slides were analyzed using fluorescence microscopy (Leica Microsystemic Inc., IL) and were photographed at 40–400x magnification.

### 2.9. Measurement of Results and Statistical Analysis

The measurement of results from images was performed using commercially available software (Northern Eclipse, version 6.0, Empix Imaging, Inc., Mississauga, ON, Canada) and Image J software (version 1.32j, National Institutes of Health, Bethesda, MD). Data from at least three samples from each subject were pooled for statistical analysis. Results are given as the mean ± standard deviation (SD). Statistical significance of any difference was calculated using Student's *t*-test, with *P* < 0.05 being considered statistically significant.

## 3. Results

### 3.1. Skeletal Muscle Atrophy Occurs in Osteosarcoma-Bearing Mice

**Figure 1:**
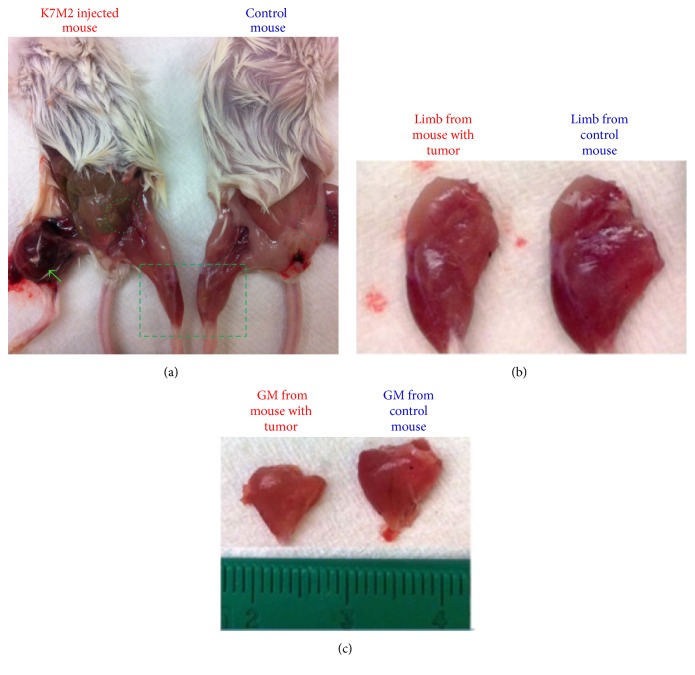
Cancer cachexia in osteosarcoma-bearing mice. (a) Six weeks after the injection of K7M2 cells into tibias of mice, tumor development can be observed in right hindlimb (green arrow). The tumor-bearing mice showed smaller muscle size (outlined with rectangle) and reduced adipose tissue (outlined with circles). (b) Left hindlimbs from mice with and without tumor. (c) Gastrocnemius (GM) muscles from mice with and without tumor.

To establish an orthotopic model of sarcoma, K7M2 murine osteosarcoma cells were injected into the right tibias of SCID/beige mice. Six weeks after K7M2 cell injection, sarcoma tumors over 1 cm in diameter were observed in the right hindlimbs of the mice (Figure 1(a), green arrow). Compared to the control mice, both the size of skeletal muscle from the uninjected left hindlimb (Figures 1(b) and 1(c)) and the volume of abdominal adipose tissue (Figure 1(a), circles) were found to be diminished in mice with tumors. The dramatic loss of muscle and adipose tissue in these mice confirm the presence of CAC.

### 3.2. Sarcoma-Bearing Mice Demonstrated Skeletal Muscle Atrophy Characterized by Smaller Myofibers and Enhanced Fibrosis

**Figure 2:**
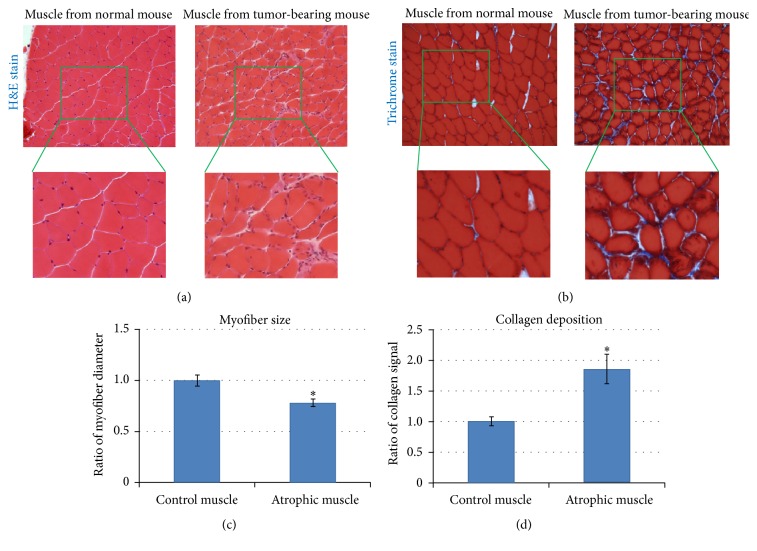
Skeletal muscle from tumor-carrying mice developed muscle atrophy. (a) H&E staining of GM muscles, revealing relative myofiber size and number of mononuclear cells (i.e., macrophages or undifferentiated muscle stem cells) in mice with and without tumor. *N* = 4 mice in each group. (b) Trichrome staining of GM muscles, demonstrating differential myofiber size and collagen deposition. *N* = 4 mice in each group. (c) Myofiber size in GM muscles. (d) Collagen deposition in GM muscles. “*∗*” in the bar chart indicates *P* < 0.05.

H&E staining and trichrome staining were performed on histologic slides of skeletal muscle tissue from tumor-bearing mice. Compared with normal mice, there was an infiltration of mononuclear cells into the skeletal muscle of tumor-bearing mice (Figure 2(a), magnified area). Also, compared to the normal mice, the myofibers within the skeletal muscle of tumor-bearing mice were smaller (Figures 2(b)(magnified area) and 2(c)) and fibrosis, reflected by collagen deposition, was increased (Figures 2(b) and 2(d)). These histologic findings corroborated the gross observation of CAC and muscle atrophy in tumor-bearing mice.

### 3.3. Notch Signaling Is Increased in Both the Tumor Tissue and Skeletal Muscle of Sarcoma-Bearing Mice

**Figure 3:**
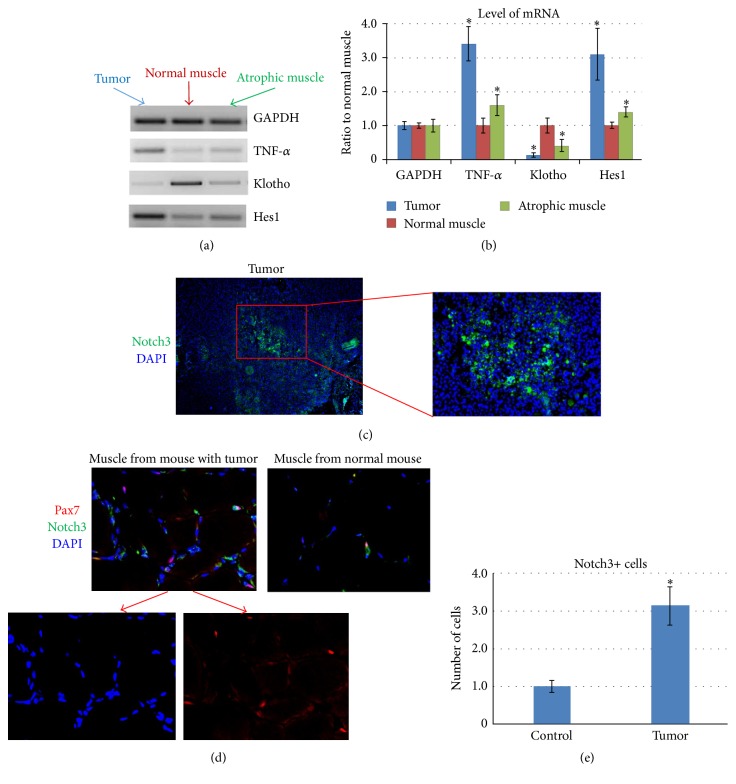
Increased Notch activation in both tumor tissues and skeletal muscle of tumor-bearing mice. (a) Semiquantitative PCR showed the gene expression of TNF-*α*, Hes1, and Klotho in tumor, normal muscle, and atrophic muscle. (b) mRNA levels of TNF-*α*, Hes1, and Klotho in 3 types of tissues. “*∗*” in the bar chart indicates *P* < 0.05 compared to normal muscle. (c) Immunostaining of tumor tissue with antibody to Notch3, showing enrichment of Notch3+ cells in tumor. (d) Immunostaining of muscle tissue with antibody to Notch3 and Pax7, showing increased numbers of Pax7+ cells, Notch3+ cells, and Pax7+/Notch3+ cells in the muscle of tumor-bearing mice. The colocations of Pax7 and DAPI are indicated with arrows in the images. (e) The quantification of Notch3+ cells in muscle of normal mice (control) and mice bearing tumor (tumor). “*∗*” in the bar chart indicates *P* < 0.05.

Semiquantitative Reverse Transcriptase-PCR (RT-PCR) was performed to compare the differential gene expression patterns of tumor, normal muscle (from mice without tumor), and atrophic muscle (from sarcoma-bearing mice). The expression of TNF-*α* and Hes1 (a downstream effector of Notch signaling) in tumor tissue was higher than both normal muscle and atrophic muscle, while the expression of Klotho [an anti-inflammatory factor [48, 49]] was lower (Figures 3(a) and 3(b)). When normal muscle and atrophic muscle were compared, the expression of TNF-*α* and Hes1 was higher in atrophic muscle, while the expression of Klotho was lower (Figures 3(a) and 3(b)). These observations suggest that both proinflammatory signaling and Notch signaling are greater in atrophic muscle compared with normal muscle.

In agreement with the observation obtained at the mRNA level, immunostaining for the Notch3 protein further demonstrated an increased number of Notch3+ cells in the tumor tissue (Figure 3(c)). Additionally, there were more Notch3+ cells in atrophic muscle when compared with normal muscle (Figures 3(d) and 3(e)). Pax7 is a cell marker for muscle stem cells (satellite cells), and NF-*κ*B-mediated enrichment of undifferentiated Pax7+ cells in muscles has been shown to promote CAC [1]. We also observed an increased number of Pax7+ cells in atrophic muscle compared with normal muscle. Some of these Pax7+ cells in atrophic muscle were also Notch3+ (Pax7+/Notch3+) (Figure 3(d)). This observation suggests that there are more undifferentiated muscle stem cells in atrophic muscle, possibly due to the activation of Notch signaling.

### 3.4. Systemic Inhibition of Notch Signaling in Tumor-Bearing Mice Reduces Muscle Atrophy and Fibrosis but Does Not Affect Tumor Size

**Figure 4:**
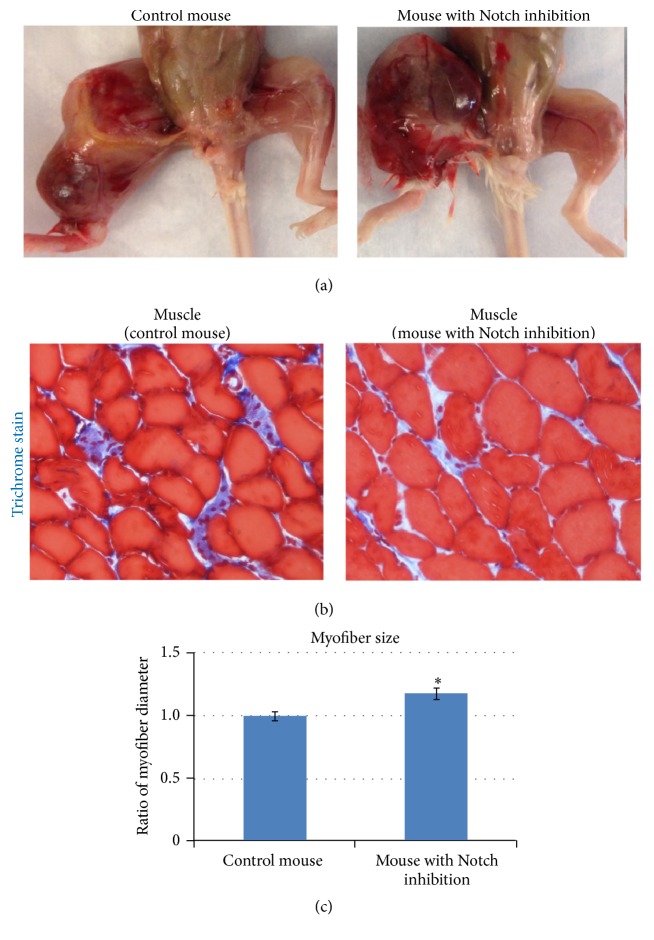
*In vivo* inhibition of Notch signaling in tumor-bearing mice reduced skeletal muscle atrophy. (a) Notch inhibitor MK-0752 (50 mg/kg) was injected starting two weeks after K7M2 cell injection, three times a week for four weeks. The size of the primary osteosarcoma tumor was unaffected by MK-0752 injection. *N* = 4 mice in each group. (b) Trichrome staining showing decreased fibrosis formation in muscles with Notch inhibition. (c) Differential myofiber size with and without MK-0752 injection. “*∗*” in the bar chart indicates *P* < 0.05.

While the effect of Notch inhibition on cancer development has been extensively studied [50–52], its effect in CAC has not been addressed. In this study, a low dose of the* in vivo* Notch inhibitor MK-0752 (50 mg/kg) [47] was injected intraperitoneally starting 2 weeks after cell injection, when tumors began to appear. Injection of MK-0752 was performed 3 times a week for 4 weeks. Results showed that the size of the primary osteosarcoma tumors was not significantly decreased by Notch inhibition (Figure 4(a)). However, trichrome staining of the muscle revealed reduced fibrosis formation (Figure 4(b)) and increased myofiber size (Figures 4(b) and 4(c)). These observations indicate that although Notch inhibition may not efficiently repress* in situ* osteosarcoma tumor growth, the muscle atrophy associated with cancer cachexia could be ameliorated by Notch inhibition.

### 3.5. Coculture of K7M2 Cells with MDSCs from Normal Muscle Yields Repressed Myogenesis of MDSCs and the Upregulation of Notch Signaling Genes

**Figure 5:**
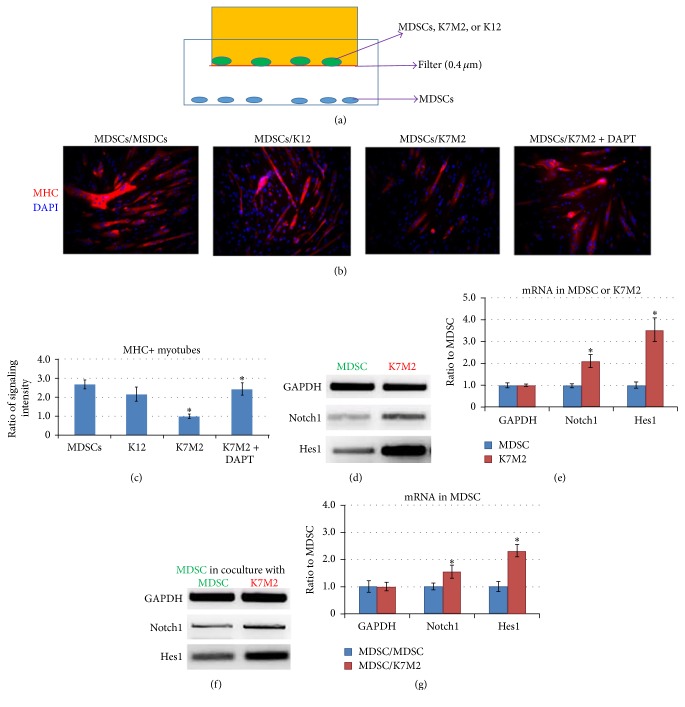
Coculture of K7M2 cells and MDSCs (from normal muscle) resulted in repressed myogenesis of MDSCs and upregulated expression of Notch genes. (a) The transwell system used in this study, including the upper chamber seeded with MDSCs, K7M2 cells, or K12 cells on the cell nonpermeable filter (0.4 *μ*m) and the lower chamber seeded with MDSCs. (b) Myogenesis (myotube formation) of cocultured MDSCs was measured by immunostaining of myosin heavy chain (MHC). K7M2 cells repressed the myogenesis potential of MSDCs, while the coadministration of DAPT could rescue the repressed myogenesis. (c) Ratio of MHC+ myotubes formed by cocultured MDSCs. *N* = 3 replicates for each group. (d) Semiquantitative PCR revealed higher mRNA levels of Notch1 and Hes1 genes in K7M2 cells, compared to MDSCs. (e) Differential expression of Notch genes in K7M2 cells versus MDSCs. (f) Semiquantitative PCR revealed higher mRNA levels of Notch1 and Hes1 genes in MDSCs cocultured with K7M2 cells, compared to MDSCs cocultured with MDSCs. (g) Differential expression of Notch genes in MDSCs/K7M2 versus MDSCs/MDSCs. “*∗*” in the bar chart indicates *P* < 0.05.

To investigate the potential influence of osteosarcoma cells on muscle stem cells, MSDCs isolated from 4-week-old control mice were cocultured with K7M2 cells, K12 cells (nonmetastatic murine osteosarcoma cells), or MDSCs themselves in a transwell system with a 0.4 *μ*m cell nonpermeable filter (Figure 5(a)). Compared with the control MDSCs (MDSC/MDSC or MDSC/K12), MDSCs cocultured with K7M2 (MDSC/K7M2) developed reduced myogenic potential, as demonstrated by the decreased immunostaining of myosin heavy chain (MHC)+ myotubes (Figures 5(b) and 5(c)). This observation indicates that tumor cells may release soluble factors that repress the myogenic differentiation of MDSCs.

To determine if Notch signaling could mediate the repressed myogenesis in MDSCs/K7M2, MDSCs cocultured with K7M2 cells were treated with the* in vitro* Notch inhibitor DAPT (*γ*-secretase inhibitor, 10 *μ*M) for 2 days and then underwent 2 days of a myogenesis assay. The myogenic potential of MDSCs/K7M2 treated with DAPT was improved compared with the MDSCs/K7M2 without DAPT treatment (Figures 5(b) and 5(c)). This observation suggests that Notch inhibition could rescue the myogenic potential of MDSCs repressed by coculture with K7M2 cells.

Our previous studies demonstrated that Notch signaling was greatly increased in K7M2 cells compared with nonmetastatic K12 cells [36]. Here, we directly compared the expression levels of Notch pathway genes between K7M2 cells and MDSCs and found they were greater in K7M2 cells (Figures 5(d) and 5(e)). We also observed that the expression of Notch pathway genes in MDSCs was upregulated upon being cocultured with K7M2 cells when compared with control MDSCs cocultured with MDSCs (Figures 5(f) and 5(g)). This observation explains the effect of DAPT treatment in rescuing the repressed myogenic potential of MDSCs (Figures 5(b) and 5(c)) and indicates that tumor cells may release a Notch-activating factor that leads to increased Notch signaling in MDSCs.

### 3.6. Exosomes from K7M2 Cells Increase Notch Activation and Repress MDSC Myogenic Potential

**Figure 6:**
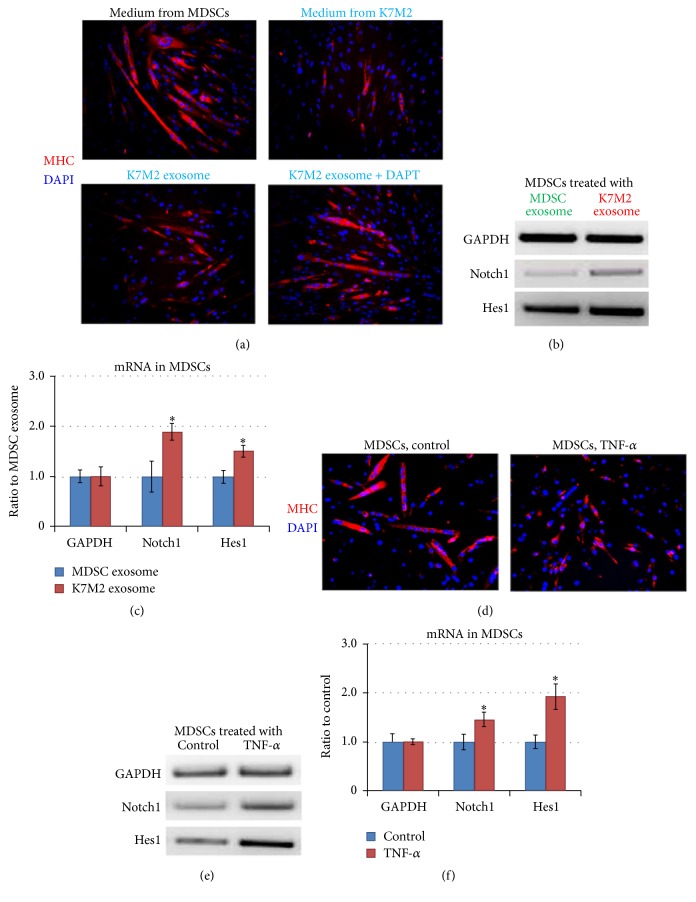
Exosomes from K7M2 cells increased Notch activation and repressed the myogenesis of MDSCs, and TNF-*α* treatment of MDSCs repressed myogenesis by activating Notch signaling. (a) MDSCs were treated with culture medium of MDSCs, culture medium of K7M2 cells, exosomes isolated from the culture medium of K7M2 cells (K7M2 exosomes), and K7M2 exosomes plus DAPT. Myotube formation was measured with immunostaining against MHC. (b) The expression of Notch1 and Hes1 in MDSCs treated with K7M2 exosomes or MDSC exosomes was compared with semiquantitative PCR. (c) Differential expression of Notch1 and Hes1 in MDSCs/K7M2 exosome versus MDSCs/MDSC exosome. (d) Myogenesis of MDSCs with and without TNF-*α* treatment was compared using immunostaining of MHC. (e) TNF-*α* treatment (20 ng/mL) of MDSCs upregulated the expression of Notch1 and Hes1. (f) Differential expression of Notch1 and Hes1 in K7M2 with or without TNF-*α* treatment. “*∗*” in the bar chart indicates *P* < 0.05.

We sought to identify the Notch-activating factors that were potentially generated and released by K7M2 cells. Exosomes released by cancer cells have been identified as important mediators of intercellular communication [37, 53]. The filter (0.4 *μ*m) used in the cell coculture system described above was permissive for translocation of exosomes (<0.1 *μ*m). To determine if exosomes might carry factors that could regulate MDSC myogenic potential, exosomes in the culture medium of K7M2 cells were isolated and added to the culture medium of MDSCs. K7M2 exosome treatment of MDSCs repressed myogenesis in a manner similar to their inhibition with K7M2 coculture (Figure 6(a)). Additionally, the expression of Notch signaling genes in MDSCs was found to be upregulated by K7M2 exosome treatment, in contrast to MSDCs treated with exosomes isolated from MDSCs (MDSC exosomes) (Figures 6(b) and 6(c)). These observations indicate that Notch-activating factors could have been delivered from K7M2 cells to MDSCs by exosomes. Further, coapplication of DAPT with K7M2 exosomes rescued the repressed myogenesis of MDSCs (Figure 6(a)). These observations indicate that exosomes from K7M2 cells may be the delivery vehicles for Notch-activating factors, which in turn upregulate Notch signaling in MDSCs and repress myogenesis.

### 3.7. TNF-*α* Treatment Increases Notch Activation and Represses the Myogenesis of MDSCs

Previous studies have revealed that proinflammatory factors, such as TNF-*α*, function as the key mediators of muscle atrophy in cancer cachexia [38–41]. Because elevated TNF-*α* expression was observed in sarcoma tumors (Figure 3), we hypothesized that another potential mechanism for Notch activation could be TNF-*α* released by the tumor into the systemic circulation exerting an effect on skeletal muscle. TNF-*α* has been found to closely interact with Notch signaling in regulating cancer development and metastasis [14, 15, 42]. Here we observed that the myogenesis of MDSCs was repressed with TNF-*α* treatment (Figure 6(d)), and TNF-*α* treatment (20 ng/mL) of MDSCs also caused the upregulation of Notch signaling genes (Notch1 and Hes1) (Figures 6(e) and 6(f)).

## 4. Discussion

The key role of Notch signaling in the regulation of skeletal muscle regeneration and stem cell function has been previously established [16–18]. The importance of Notch signaling in mediating denervation-induced muscle atrophy is also well documented [54, 55]. However, although skeletal muscle atrophy is the key feature of CAC, the potential role of Notch in skeletal muscle biology and stem cell function in CAC is still unknown. Notch activation in the stem cell niche is known to mediate the quiescence of muscle stem cells in skeletal muscle, which is important for maintaining the integrity and function of the stem cell pool. However, constant activation of Notch signaling adversely affects muscle regeneration and the downregulation of Notch signaling is preferred during certain stages of muscle regeneration [24, 56]. This study is the first attempt to understand the role of Notch signaling in cancer-associated cachexia.

We have previously shown that the osteosarcoma cell line K7M2 actively expresses Notch genes (Notch1, Notch2, and Notch4; Hes1), but not Notch3 [36]. In the described coculture study of MDSCs and K7M2 cells, the increased expression of Notch1 and Hes1 in the MDSCs was found to correlate with the repressed myogenic potential of the cells (Figure 5(f)), while the expression of Notch3 was not obviously changed (data not shown). Although western blot was not performed to confirm the production of Notch1 and Notch3 proteins, the increased expression of Hes1 (a key downstream Notch effector) indicates that the overall Notch signaling in MDSCs was increased through coculture with K7M2 OS cells. Future studies will evaluate Notch1 Intracellular Domain (NICD) expression and protein production, as well as Hes1 expression, which could build an even stronger case for our hypothesis. This current result also reveals the coactivation of proinflammatory signaling and Notch signaling in both the K7M2-induced osteosarcoma tumor and the atrophic muscles of tumor-bearing mice (Figure 3). Proinflammatory factors, such as TNF-*α*, have been shown as key mediators of cancer cachexia [38–41]. Close correlation of TNF-*α* with Notch in the regulation of cancer development and metastasis has also been described [14, 15, 42]. TNF-*α*/NF-*κ*B can activate Notch by inducing Jagged1 expression, and Notch activation in turn could sustain excessive proinflammatory signaling [13, 14, 57, 58]. However, the interaction of Notch signaling and TNF-*α*/NF-*κ*B signaling in CAC has not been described. In this study, we have observed that the atrophic muscles in sarcoma-bearing mice feature the upregulated expression of both Notch genes and TNF-*α*, while the expression of anti-inflammation factor Klotho was downregulated (Figure 3). Therefore, we suggest that TNF-*α* may have circulated from the tumor to skeletal muscle and interfered with muscle stem cell activity and muscle regeneration via interaction with Notch signaling.

In addition to proinflammatory factors (e.g., TNF-*α*), our results indicate that exosomes from tumor cells may also serve to activate Notch signaling in the skeletal muscle. Previous studies have demonstrated that microRNAs (miRNAs) play an important role in exosome-mediated intercellular communication in cancer cells [59, 60]. MicroRNAs have been recently recognized to play critical roles in the Notch signaling pathway, and crosstalk between miRNA and Notch signaling pathways in tumor development has been demonstrated [61]. Candidate miRNAs that could mediate Notch-activating signaling may include miRNA199b-5p [62] or miRNA-21 [63]. The delivery of Notch ligand DLL4 via exosomes has also been demonstrated as a novel mechanism for Notch ligands to expand their signaling potential beyond cell-cell contact [64]. Therefore, we suggest that exosomes from K7M2 cells may contain miRNAs, Notch ligands (e.g., DLLs or Jagged), or both, allowing transfer of the Notch-activating signal from tumor to muscle.

Strategies to therapeutically modulate Notch signaling have been of great interest in the research and treatment of cancer. Notch inhibitors, including *γ*-secretase inhibitors, have been extensively studied in clinical trials in patients with solid tumors [51, 52, 65]; MK-0752, a potent inhibitor of *γ*-secretase, has been utilized in clinical trials to study its effect on Notch inhibition and cancer development [45, 46]. Our current study illustrated that although systemic MK-0752 treatment of tumor-carrying mice at a lower dosage may not efficiently repress the gross development of osteosarcoma, it could still improve the histology of atrophic muscle. The systemic effect of MK-0752 on osteosarcoma metastasis is currently under investigation.

## 5. Conclusion

Our current results demonstrate that Notch signaling is overactivated in the skeletal muscle of sarcoma-bearing mice and is involved in the development of muscle atrophy.* In vitro* studies further reveal that Notch-activating signals could be transferred from tumor cells (K7M2) to muscle stem cells (MDSCs) via exosomes or TNF-*α* released by the tumor cells. Our results reveal a novel role for Notch signaling in the mediation of skeletal muscle atrophy in CAC. Therefore, in addition to the role of Notch signaling in cancer development and metastasis, the role of Notch in cancer cachexia should also be further investigated.

## Abbreviations

MDSCs: Muscle-derived stem cells
CAC: Cancer-associated cachexia
SAC: Sarcoma-associated cachexia
f-MHC: Fast-myosin heavy chain
NICD: Notch1 Intracellular Domain.
